# More than a simple fixed action pattern: Yawning in drills

**DOI:** 10.1007/s10329-024-01127-7

**Published:** 2024-04-22

**Authors:** Alice Galotti, Giulia Fausti, Grazia Casetta, Andrea Paolo Nolfo, Veronica Maglieri, Elisabetta Palagi

**Affiliations:** 1 Unit of Ethology, Department of Biology, University of Pisa, Via Alessandro Volta 6, 56126 Pisa, Italy; 2 Natural History Museum, University of Pisa, Via Roma 79 Calci, 56011 Pisa, Italy

**Keywords:** *Mandrillus leucophaeus*, FACS, Spontaneous yawning, Behavioral state changing, Yawn response

## Abstract

In the last decade, increasing attention has been devoted to exploring some aspects of yawning in non-human animals. With their chin red mark, bony paranasal swellings, male large brains and long canines, drills (*Mandrillus leucophaeus*) offer a robust model for testing hypotheses on the phenomenon. We identified two yawn variants (covered, Y_CT_ and uncovered teeth, Y_UCT_) which differ in terms of recruitment of muscular action units (AUs). We tested the effects of several variables (sex, dominance rank, context) on the duration of the yawn and the probability of Y_CT_ or Y_UCT_ occurrence. We found that males performed longer and more Y_UCT_ than females. These findings support the *Brain Cooling Hypothesis* suggesting that those species showing large brains tend to display larger and longer yawns. We also tested the *State Changing Hypothesis* predicting the presence of a temporal association of yawning and ongoing behavioral transitions. A sequential analysis revealed that after 30 s following a yawn, drills were significantly more likely to change their behavioral state. Through the observation of yawning, conspecifics might gain knowledge of impending state changes. Seeing other’s yawns increased the probability of a similar response in the observers, thus suggesting the presence of yawn contagion in drills. Although the dataset needs to be expanded, our findings indicate that yawning is variable in drills, it can be associated with subjects’ state changes, and the imminent shifts can be perceived/processed by conspecifics.

**Graphical Abstract:**

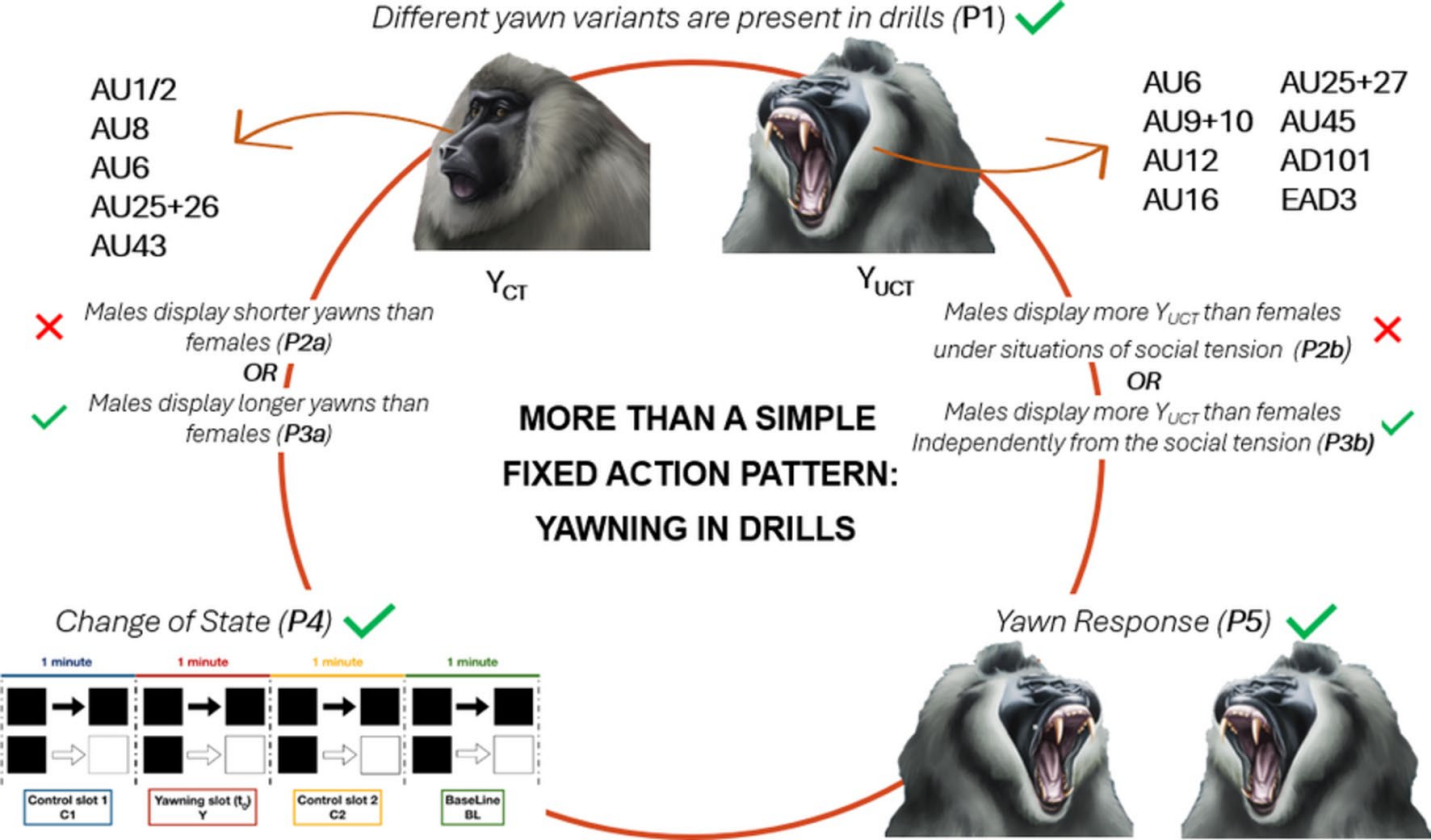

**Supplementary Information:**

The online version contains supplementary material available at 10.1007/s10329-024-01127-7.

## Introduction

Despite its simplicity in the motor execution and ubiquity across vertebrates, a number of hypotheses have been formulated over the centuries to explain the function of spontaneous yawning (Gallup 2022; Matikainen and Elo 2008; Provine 2005; Schiller 2002). In 1986, summarizing the concept, Provine and Hamernik (1986, p. 120) stated that “yawning may have the dubious distinction of being the least understood common human behavior”. Yawning may seem to be morphologically similar across all vertebrate taxa (Casetta et al. 2022; Deputte 1994; Walusinski and Deputte 2004). In mammals, three different phases can be distinguished during a yawning event (Barbizet 1958). The sequence starts with a slow and wide mouth opening accompanied by a deep inhalation, until a maximum mouth opening is reached, then a quick closure of the mouth and a short exhalation occur. Other motor actions such as eye closures, vocalizations, pandiculation, and even tongue protrusion can accompany yawning in different species (Palagi et al. 2020). Moreover, a certain level of variability can also be found in the duration of the yawning motor action (Massen et al. 2021).

Although difficult to disentangle, the functions that have been attributed to yawning can be based on the different physiological states experienced by the yawner (*Physiological* domain) and the social contexts in which a yawn occurs (*Social* domain) (Guggisberg et al. 2010)*.* The hypotheses included in the *Physiological* domain are based on the assumption that yawning plays a role in regulating specific bodily activities such as blood oxygen levels in the brain, drowsiness, and thermoregulation (Deputte 1994; Gallup 2022; Gallup and Eldakar 2012; Zilli et al. 2008; Guggisberg et al. 2010; Miller et al. 2010). Several studies show that yawning cools the body and brain, making them sensitive to the variations of the environmental temperature (Gallup 2011; Gallup and Eldakar 2012; Shoup-Knox et al. 2010; Massen et al. 2021).

The *Drowsiness Hypothesis*, predicts that yawning is a mechanism linked to changes in the state of alertness due to increased frequency during relaxed periods characterized by an alternation of resting and sleeping phases (humans, *Homo sapiens* Giganti et al. 2010, Greco et al. 1993, Provine 2005; South American sea lions, *O. flavescens,* Palagi et al. 2019a, b; ostrich, *Struthio camelus australis*, Sauer and Sauer 1967; African elephant, *Loxodonta africana*, Rossman et al. 2017; spotted hyaenas, *Crocuta crocuta*, Casetta et al. 2022).

The hypotheses included in the *Social* domain are based on the assumption that yawning is sensitive to the stimuli an animal receives from its social environment (Baenninger 1997). According to the *Social Distress Hypothesis,* a socially stressful event can increase the probability of yawning concurrently with other self-directed behaviors (e.g., self-scratching and self-grooming) thus helping the subject to restore emotional homeostasis (Palagi et al. 2019a, b). In primates, self-directed behaviors tend to be frequently performed under situations of psychosocial stress with their rates being influenced by the administration of anxiogenic and anxiolytic substances (Maestripieri et al. 1992). The relation between anxiety state and yawning has been reported in many different taxa such as birds (budgerigar, *Melopsittacus undulatus*, Miller et al. 2010), rats (*Rattus norvegicus,* Moyaho and Valencia 2002), South American sea lions (*Otaria flavescens*, Palagi et al. 2019a, b) and several primate species (*Theropithecus gelada*, Leone et al. 2014; *Lemur catta*, *Propithecus verreauxi*, Zannella et al. 2015). The so-called “threat yawns” (sensu Altmann 1967), which differ from those observed in relaxed contexts (Leone et al. 2014), are mostly present under situations of conflict and social tension. In most Old World monkey species, males exhibit a strong sexual dimorphism in the canine size and yawns showing the long teeth are often emitted under competitive contexts (Hadidian 1980; Redican 1975). Several authors agree on the fact that the exposure of canines through large yawns may be a signal conveying messages of threat and arousal (Altmann 1967; Deputte 1994; Zannella et al. 2017, 2021). As a whole, if we look at the increasing literature devoted to clarifying the possible functions of yawning, it appears evident that the classical dichotomous classification of its functions in the *Physiological* and *Social* domains is crumbling with a more holistic view that seems to better fit with the explanation of the phenomenon.

Despite its morphological consistency across different taxa, the different combinations of motor actions involved in a single yawning event suggest a certain degree of variability of the phenomenon (Gallup et al. 2016; Guggisberg et al. 2010; Provine 2012; Massen et al. 2021; Walusinski and Deputte 2004). One of the most evident elements of yawning diversity is the visibility of both upper and lower canines as reported for several monkey species (Baenninger 1997; Deputte 1994; Leone et al. 2014). For example, in macaques, yawning can involve different degrees of mouth opening (covered teeth and uncovered teeth yawning, Zannella et al. 2017, 2021). In particular, in Tonkean macaques, under social tense situations males perform very short and uncovered teeth yawns (Zannella et al. 2021). The authors suggested that these short versions of yawning can function as displays conveying a threatening message at least in this tolerant Sulawesi species of macaques. Therefore, there is increasing evidence showing that the morphology of a yawn can indirectly inform its potential functions by contextualizing the yawning event. In chimpanzees, covered teeth yawning is often associated with situations producing anxiety in the subject (Vick and Paukner 2010). In contrast, in geladas, covered teeth yawning appears to be expressed under relaxed contexts (Leone et al. 2014). Some authors argue that variability in yawn morphology can be related to the different levels of tolerance expressed by some primate species (geladas, Leone et al. 2014; Palagi et al. 2009; macaques, Dobson 2012; Maestripieri 1999; Zannella et al. 2017).

An association between yawning and behavioral transitions (*State Changing Hypothesis*), even occurring outside the awaking/sleeping context, has been highlighted in several primate (humans, Giganti and Zilli 2011; Provine 2005; geladas, Leone et al. 2014;* Propithecus verrauxi* and* Lemur catta*, Zannella et al. 2015) and non-primate species (*Panthera leo*, Casetta et al. 2021; *Crocuta crocuta*, Casetta et al. 2022) probably indicating a shift in the motivational state of the subject.

A further important feature linking yawning to social environment is the susceptibility to respond to conspecifics’ yawn with another yawn (yawn contagion) (Provine 2005). Yawn contagion has been extensively documented in human (Norscia and Palagi 2011; Palagi et al. 2020; Provine and Hamernik, 1986; Provine, 2005) and nonhuman animals (birds, Miller et al. 2012; social carnivores, Romero et al. 2014; Casetta et al. 2021; Ake and Kutsukake 2023; monkeys, Palagi et al. 2009; Valdivieso-Cortadella et al. 2023; great apes, Campbell and Cox 2019; Demuru and Palagi 2012) with some exceptions (birds, Gallup et al. 2022; lowland gorillas, Palagi et al. 2019a, b). It has been recently demonstrated that yawn contagion can also occur between different species (Gallup and Wozny 2022; Pedruzzi et al. 2022).

Here, we aim at exploring some aspects of yawning in the second largest European zoo-housed group of drills (*Mandrillus leucophaeus*). Due to its relevant social propensity (Gartlan, 1970) and strong canine sexual dimorphism (Marty et al. 2009), the drill is an excellent model to test hypotheses on the variability and distribution of yawning according to some individual intrinsic characteristics and social contexts. If, as it occurs in other primate species (Baenninger 1997; Deputte 1994; Vick and Paukner 2010; Zannella et al. 2021), different types of yawns are present in drills, we expect that such variability (covered teeth yawns, Y_CT_; uncovered teeth yawns, Y_UCT_) can be demonstrated by the quantitative analysis of the recruitment of muscular action units through Facial Action Coding System (FACS) (Prediction 1).

The *Sexual Dimorphism Hypothesis* states that in those species, in which males possess larger canine size than females, males tend to expose teeth during short yawns (geladas, Leone et al. 2014, macaques, Zannella et al. 2017; Tonkean macaques, Zannella et al. 2021) especially under social tension conditions (*Social Distress Hypothesis*). If Y_UCT_ have a role in expressing the arousal state in drills, we expect that both social context (tension/relax) and sex of the yawner can influence yawn morphology and duration. In particular, we predict that males display shorter Y_UCT_ than females and that Y_UCT_ (see Figure S1) are mainly performed under situations of social tension (Prediction 2a and 2b).

Given the greater thermolytic needs of larger brains, the *Brain Cooling Hypothesis* posits that subjects with larger brains would yawn longer to achieve comparable cooling effects (Gallup et al. 2016; Massen et al. 2021). Since in drills, as in other highly dimorphic monkey species, the sexual dimorphism is also highly evident in the brain dimensions (Osman-Hill 1970, 1974), if yawning has a role in regulating brain temperature, we predict to find longer and larger yawns (Y_UCT_) (Prediction 3a) in males than in females independently from the context in which the behavior occurs (Prediction 3b).

If the *State Changing Hypothesis* (Provine 2005) explains the occurrence of spontaneous yawn in drills, we predict that yawning mainly occurs in association with behavioral transitions (Prediction 4). Finally, if animals are susceptible to others’ yawns, we expect that seeing others’ yawn increases the probability of a yawn response in the receiver (Prediction 5).

## Materials and methods

### Subjects and data collection

We collected behavioral data on a group of 13 drills (*Mandrillus leucophaeus*) at the Dvůr Králové Zoo (Czech Republic). See Table 1 for details of group composition. Kinship between subjects was known. The enclosure included an outdoor (about 1600 m^2^) and an indoor facility (about 50 m^2^). The animals were free to move in the outdoor and in the indoor facilities. Both facilities were provided with environmental enrichments that were frequently renovated to keep animals active and guarantee their welfare. Animals were trained only for medical purposes. Abnormal behaviors were never observed during the data collection. Drills had access to food, mostly fruit and vegetables, every day from 8:00 to 11:00 am and water was available ad libitum.

**Table 1:** The group composition, total observation time expressed in minutes, and number of Y_CT_ and Y_UCT_ for each individual

Subjects	Sex	Age in years	ADI_values_	Total observation time (min)	No. of Y_CT_	No. of Y_UCT_	#Y_CT_/h	#Y_UCT_/h
Chepo	♂	18 (adult)	0.9950	1713	61	130	1.07	2.28
Kumasi	♀	14 (adult)	0.8153	1252	12	8	0.29	0.19
Ricardo	♂	15 (adult)	0.7998	1114	17	57	0.46	1.54
Kebale	♀	15 (adult)	0.4857	1015	2	3	0.06	0.09
Ndolo	♂	4 (juvenile)	0.4090	456	2	3	0.13	0.20
Kwai	♀	3 (juvenile)	0.3333	579	2	0	0.11	0.00
Mambilla	♂	4 (juvenile)	0.3000	434	4	4	0.27	0.27
Efuru	♀	3 (juvenile)	0.2213	667	0	0	0.00	0.00
Mumba	♂	2 (juvenile)	0.2150	567	2	7	0.11	0.37
Obudu	♂	4 (juvenile)	0.1833	383	2	3	0.15	0.23
Kara	♀	3 (juvenile)	0.1780	733	1	0	0.04	0.00
Atu	♂	1 (infant)	*	848	2	1	*	*
Kali	♀	0.5 (infant)	*	552	1	0	*	*

The* asterisks* indicate the missing values for the frequencies and the values of ADI for infants (not included in the analyses)

ADI_values_ values obtained via Average Dominance Index procedure, *Y*_*CT*_ yawn with covered teeth, *Y*_*UCT*_ yawn with uncovered teeth, # number, *h* hours of observation

The age categorization was done on the basis of Setchell et al. (2005)

The observational period lasted 2 months (August 14–October 15, 2020). The animals were filmed 5 days a week in the outdoor/indoor facilities according with the weather conditions. Multiple opportunistic viewpoints were accessible to maximize group observability (outdoor facility: blind spots < 10% of the total area; indoor facility: blind spots < 5% of the total area). Following the natural dynamics, the entire group split into subgroups of individuals. When such subgroups became well visible to the observers, they were opportunistically identified as focal subgroups. We tried to optimize our observational efforts with the goal of obtaining a comparable observation time for each animal. Due to the naturalistic conditions, it was not possible to achieve comparable observation time across all the individuals, considering that some of them were generally less visible (see Table 1 for the exact hours of observation per subject). Each observation day lasted about 8 h spanning morning and afternoon (8:00 am to 6:30 pm). The observation time slots were decided at daily level to avoid short periods characterized by the highest presence of visitors. At the end of the data collection, such a procedure allowed us to cover the entire time window (8:00 am to 06:30 pm). The first 10-day slot of observations was used to habituate animals to the presence of the operators and cameras, although the drills were already accustomed to the passage of people. No data collection occurred during this time slot. Subjects were identified by sex, age, and their external features (e.g., fur color, scars, and facial traits). The full HD video cameras (Canon EOS 110 D; Panasonic Lumix FZ 82) were always mounted on a stand to guarantee a high video quality. At the end of the observational period, we collected 732 videos for a total of 170 h. The duration of each video was not predetermined but decided opportunistically. When a subgroup separated, the operators interrupted the video and started a new one focusing on another subgroup. This resulted in a range of 5–20 min per video, with an average duration of 12.43 min ± 4.350 SD.

### Video analyses and operational definitions

Five operators were involved in the study (four of them are co-authors). Two of them collected the videos (GC, APN), four of them checked the videos (AG, GF, GC, APN), and three of them (the certified coders, AG, GF, and an assistant) performed the FACS analysis.

Videos were analyzed via Pot-Player software that allows slowing down the frames of interest (yawning slowed down to 70%). We used the all-occurrences sampling method (Altmann 1974) to obtain all the yawning events from the videos.

Here, the following operational definitions are discussed: recording of spontaneous yawning events; recording of aggressive events; definition of aggressive events; analysis of yawn variants using FACS; definition of context, behavioral state change and yawn response; inter-observer reliability.

A yawn was defined according to the following criteria: deep inhalation, brief peak with apnea followed by a short exhalation, an active jaw opening and passive jaw closure, possibly eye closure, sometimes accompanied by neck/head tilting, tongue protrusion, scalp retraction, and pandiculation. For the yawning events, the Cohen’s κ reached the 1.00 score for each dyad of observers.

For each spontaneous yawn event we listed: (i) the identity of the yawner, (ii) duration of the yawning event (seconds), (iii) the visibility of canines (covered, Y_CT_; uncovered teeth yawns, Y_UCT_), (iv) the exact time of the day, and (v) the yawner’s posture (defined as lying, sitting, standing, walking), (vi) the context (resting-sleeping/tension). All the events were analyzed frame-by-frame (accuracy 0.02 s). A yawn was considered to start in correspondence with the first frame in which the lips appeared parted and to end in the correspondence with the frame in which the lips appeared closed.

In addition to yawning events, we also collected aggressive events, which were subsequently used to calculate the Average Dominance Index (ADI) for each subject (see below for details).

#### Definition of the aggressive events

We classified the dyadic aggressive events according to their intensity (contact vs. no contact/threats interactions). We selected only those aggressive encounters in which the winner and the victim of aggression were clearly discernible through the presence of submissive/fear signals.

#### Analysis of the yawn variants

FACS (Facial Action Coding System) is an observational scientific tool allowing an objective measurement of facial movements in human and non-human animals (humans, Ekman and Friesen 2003; macaques, Parr et al. 2010; dogs, Waller and Micheletta 2013). The AUs emerge from the single or combined contraction of facial muscles producing external facial changes.

To codify the AUs recruited during the yawning events, we drew from different resources. We used the human FACS adapted by Dobson (2009) for non-human anthropoids, as it has been successfully done for geladas (*Theropithecus gelada*) (Lazow and Bergman, 2020). We also used the MaqFACS for the AUs (AU6, AU25, AU26, AU27, AU43, AU45, EAD3, and AD101) not included by Dobson (2009), which we codified in drills. Moreover, we verified that the muscle groups underlying the AUs, recruited during yawning, described for *Theropithecus,* were also present in *Mandrillus* spp. (Osman-Hill 1970, 1974). The anatomical comparison of facial muscles at the basis of the activation of the different units makes us confident that we can apply FACS (Dobson 2009) and MaqFACS (Parr et al. 2010) to our species of interest. We quantified the different AUs recruited in the two types of yawns already identified in literature for monkeys (Baenninger, 1997; Deputte 1994; Zannella et al. 2015, 2021), excluding the AUs describing head movements (see Table 2).

**Table 2:** FACS adaptation for drills

AU code	AU description	Muscles recruited	Species and references
AU1 + 2	Brow raiser	*Frontalis* (medial and lateral parts)	*M. rhesus*, Dobson (2009)
AU6	Check raiser	*Orbicularis oculi*	*M. rhesus*, Parr et al. (2010)
AU8	Lips towards each other	*Orbicularis oris*	*M. rhesus*, Parr et al. (2010)
AU9 + 10	Nose wrinkle and upper lip raiser	*Levator labii superiorisalaeque nasi*	*M. rhesus*, Dobson (2009)
AU12	Lip corner puller	*Zygomatic major*	*M. rhesus*, Dobson (2009)
AU16	Lower lip depressor	*Depressor labii inferioris*	Macaque, Dobson (2009)
AU25 + 26	Lips parted and jaw drop	*Mylohyoid in the neck, depressor anguli oris, levator labii inferioris*	*M. rhesus*, Parr et al. (2010)
AU25 + 27	Lips parted and mouth stretch	*Mylohyoid in the neck, depressor anguli oris, levator labii inferioris*	*M. rhesus*, Parr et al. (2010)
AU43	Eye closure	*Levator palpebrae superioris*	*M. rhesus*, Parr et al. (2010)
AU45	Eye blinking	*Levator palpebrae superioris, orbicularis occuli*	*M. rhesus*, Parr et al. (2010)
AD101	Scalp retraction	*Platysma(?)*	*M. nigra,* Clark et al. (2020)
EAD3	Ears flattener	*Posterior auricularis*	*M. rhesus*, Parr et al. (2010)

Action Units and Action Descriptor observed during yawning in* Mandrillus leucophaeus*

The frame sequences of Y_CT_ and Y_UCT_ were compared with a neutral face whenever possible and screened at least twice before assigning the AUs. Both for Y_CT_ and Y_UCT_, the determination and number of each AU were assigned to the frame of mouth gaping peak. The first author codified the AUs of each yawning event. To check for inter-coder reliability, three MaqFACS certified coders (AG, GF, and a field assistant passed the certification tests) analyzed 25 Y_CT_ (from eight individuals) and 39 Y_UCT_ (from eight individuals) independently.

The yawns were coded using all the AUs described in Table 2 without discarding any of them a priori; nevertheless, not all the AUs were associated with each yawning event. Considering both Y_CT_ and Y_UCT_, we detected the involvement of a total of ten AUs, one Action Descriptor (AD), and one Ear Action Descriptor (EAD). The AUs considered in the statistical analyses were: AU1/2 = brow raise; AU6 = cheek raiser; AU8 = lips towards each other; AU9 + 10 = nose wrinkle and upper lip raiser; AU12 = lip corner pull; AU16 = lower lip depression; AU25 = lips part; AU26 = jaw drop; AU27 = mouth stretch, AU43 = eye closure; AU45 = eye blink (Parr et al. 2010). The only EAD coded was ears flattened (EAD3) (Parr et al. 2010) and the AD was the scalp retraction (AD101) (Correia-Caeiro et al. 2021). Recently, Clark et al. (2020, p. 414), included the code AD101 in a FACS adaptation for *Macaca nigra*, by giving the following description: "The hair on the top of the head, including the crest, flattens as the skin is pulled backward. Skin on the forehead and temples appears stretched.” After the analysis of several yawning events, we decided to include the AD101 in the FACS adaptation that we did for our species of interest with the same description reported by Clark et al. (2020). For both Y_CT_ and Y_UCT_, the determination and number of AUs were assigned to the frame relative to the peak of the facial expression. Every observed yawn contained only one peak.

#### Definition of the context

Yawns were also classified according to the contexts of the yawner (resting-sleeping/tension) (Zannella et al. 2021). During the resting/sleeping context, the animal was not involved in any social interaction, and remained lying down, shifting from an awake to a sleeping phase. During the social tension phase, the subjects were involved in or witnessed an aggressive/threatening interaction. The phase included the duration of the whole aggressive/threatening interaction and the 3-min time block following such event. We did not take into account contexts that were not clearly classifiable into the two categories under consideration.

#### Behavioral state change

For comparative purposes, to test the *State Changing Hypothesis* we applied the same procedure already adopted by Casetta et al. (2022). To understand whether a yawning event modified the probability for the animal to change its behavioral status, we considered four different time slots: 1-min yawn slot (Y) including the yawning event occurring at t_0_; 1-min control slot (C_1_) immediately preceding the Y slot; 1-min control slot (C_2_) immediately following the Y slot; 1-min baseline slot (BL) (Figure S2). The BL was obtained by selecting in the same day, a 1-min block of observation on the same animal showing the same behavioral state (lying down, standing, walking, or sitting) recorded for Y slot and in absence of yawning. For each time slot, we verified the presence/absence of behavioral state shifting (e.g., sitting/standing, standing/sitting, walking/standing, walking/sitting). The Y slot lasted 1 min divided into 30-s immediately before and 30-s immediately after the yawning event (Figure S2). This tight time window limited the possibility that the behavioral state change was not linked to the yawning event. Consequently, we defined the other slots by using the same 1-min time approach to ensure the same probability for a state change to occur.

#### Definition of yawn response

We never recorded any vocalization during yawning*.* After the first yawning event (stimulus) emitted by an individual (trigger), all the subjects present in the video were observed for the following 3 min. Subjects were divided into two groups: subjects who could see the yawning stimulus (Observers) and subjects who could not see it (Control individuals). All the yawning responses were recorded for category of subjects. A yawn was considered to be seen when there were no visual barriers separating the receiver and the first yawner and when the receiver was positioned to see the head of the first yawner. All yawns emitted by the receiver after seeing the previous yawn were considered as responses if they occurred within 3 min after the perception of the triggering stimulus. The probability of coding a spontaneous yawn as a response yawn is lower in the first 3 min after the perception of the yawning stimulus than later, when autocorrelation is more probable. Actually, the presence of a yawn performed by a subject at t_0_ increases the probability to have another yawn by the same subject at t_(0+X)_ where X is the increasing unit of time (Campbell and Cox 2019). For this reason and comparative purposes, we adopted the 3-min time window criterion to record the yawn response (*Canis lupus*, Romero et al. 2014, *Gorilla gorilla gorilla,* Palagi et al. 2019a, b, *Pan troglodytes*, Campbell and Cox 2019; *Pan paniscus, Homo sapiens*, Palagi et al. 2014, *Panthera leo*, Casetta et al. 2021, 2022). All the yawns occurring after seeing others’ yawns (within 3 min) were considered as responses and consequently excluded from the analysis regarding spontaneous yawning.

#### Reliability

The inter-observer reliability was calculated during the entire course of the analysis at regular intervals (about every 35 h of video analyzed; *N* = 5 checks) on the 15% of videos collected (25.5 h) that were randomly selected and independently analyzed by each of the four observers (AG, GF, GC, APN).

For each configuration of yawn obtained by FACS, we calculated the reliability via the following equation (recommended by the human FACS manual, Ekman et al. 2002):

$$ \frac{{{2}\;{\text{(number of AUs agreed by both coders)}}}}{{\left( {\text{number of AUs coded by coder 1}} \right)\,{ + }\,{\text{(number of AUs coded by coder 2)}}}} $$

This formula calculates the agreement for each expression ranging from 0 to 1 (0 = no agreement; 1 = total agreement). The average agreement for yawns analyzed via FACS was 0.94 for AG-GF dyad, 0.98 for AG-field assistant dyad, and 0.96 for GF-field assistant dyad.

For the occurrence of aggressive/threatening interactions the agreement (Cohen’s κ, Cohen 1960) between the different dyads of observers was calculated at the beginning and at the end of the video analysis (AG-GF: 0.770–0.762, AG-GC: 0.860–0.857, AG-APN: 0.856–0.810, APN-GC: 0.874–0.920, APN-GF: 0.892–0.870, GC-GF: 0.839–0.835).

For the behavioral state changes of the subjects the inter-observational agreement (Cohen’s κ, Cohen 1960) was calculated at the beginning and at the end of the video analysis (AG-GF: 0.910–0.918, AG-GC: 0.887–0.900, AG-APN: 0.912–0.919, APN-GC: 0.889–0.918, APN-GF: 0.890–0.920, GC-GF: 0.894–0.905). As for the time slot in which the state changed or not, the reliability assessment provided a total agreement (100%) between all the different dyads of observers.

For the condition seen/not-seen of the previous yawns we obtained the following values of Cohen’s κ (AG-GF: 0.843–0.886, AG-GC: 0.852–0.872, AG-APN: 0.839–0.901, APN-GC: 0.801–0.863, APN-GF: 0.822–0.871, GC-GF: 0.837–0.879).

### Data analyses and statistics

*Different yawn variants are present in drills* (Prediction 1).

To quantitatively demonstrate the difference between the two configurations (Y_CT_ and Y_UCT_), we applied a “back-and-forth” methodological approach. As a first step, we classified a priori Y_CT_ and Y_UCT_ according to the existing literature (Zannella et al. 2017, 2021). As a second step, via FACS properly adapted for nonhuman anthropoids (Dobson 2009) and for macaques (Parr et al. 2010; Waller and Micheletta 2013), we codified the Action Units (AUs) recruited for each yawning event. Finally, we submitted the AUs to a hard-clustering analysis by applying an unsupervised k-means algorithm.

To quantify the optimal number of yawning morphs and reasonably divide our dataset according to the different combinations of the AUs recruited, we applied principal component analysis of mixed data (PCAmixdata package in R; Chavent et al. 2014) to the string of AUs constituting each yawning event. This analysis uses a generalized singular value decomposition (GSVD) of pre-processed data. The GSVD includes standard PCA and multiple correspondence analysis (MCA) as special cases, allowing it to extend standard multivariate analysis methods to incorporate categorical data (Chavent et al. 2014). To confirm the a priori classification of the yawning morphs in covered (Y_CT_) and uncovered teeth (Y_UCT_) (Baenninger 1997; Deputte 1994; Zannella et al. 2015, 2021), we used a k-means unsupervised clustering by one-hot encoding the data to group the cases and visualize data (Hartigan and Wong 1979). We one-hot encoded the data by creating dummy variables for each value of the category and by converting the categorical variable into a one-hot vector representation. Since the PCA identified two data groups, we performed a k-means clustering on the one-hot encoded data with *k* = 2. Via the Fisher’s test, we verified which AUs were significantly different between Y_CT_ and Y_UCT_.

#### Calculation of the Average Dominance Index (ADI)

The steepness of the dominance hierarchy was obtained from square-matrices of decided conflicts (i.e., all conflicts in which the victim and the winner were clearly discernible) (via Average Dominance Index method (ADI, Saccà et al. 2022). Other methods (e.g., normalized David’s score) have the disadvantage of having a lower slope when there are high proportions of unknown relationships (dyads without agonistic interactions). The ADI (the average proportion of wins by each individual from all its opponents) reduces the bias due to unknown relationships by excluding them. The ADI values for each subject are reported in Table T1.

#### Males display shorter yawns than females (MODEL_duration_, Prediction 2a) OR longer yawns than females (MODEL_duration_, Prediction 3a)

To investigate which factors affected the DURATION of the yawn, we ran a linear mixed model (LMM; glmmTMB R-package; Berry et al. 2017; Kuhn et al. 2020; Version 1.4.1717). The logarithm of the DURATION was the response variable (Gaussian distribution, N_observations_ = 319). We verified the normal distribution and homogeneity of the model’s residuals by looking at the Q-Q plot and plotting the residuals against the fitted values. The fixed factors were the CONTEXT (resting-sleeping/tension), the SEX of the yawner (male/female), the ADI values (dominance rank), the DAYTIME (8:00–10:00 am; 10:00–12:00 am; 12:00 am to 2:00 pm; 2:00–4:00 pm; 4:00–6:00 pm) and the MORPHOLOGY (Y_CT_/Y_UCT_). No collinearity was found between the fixed factors (range VIF_min_ = 1.02; VIF_max_ = 1.07). The yawner identity (ID yawner) was included as a random factor.

#### Males display more Y_UCT_ than females under situations of social tension (MODEL_morphology_, Prediction 2b) OR independently from situations of social tension (MODEL_morphology_, Prediction 3b)

To investigate which factors affected the MORPHOLOGY of the yawn, we ran a generalized linear mixed model (GLMM; glmmTMB R-package; Berry et al. 2017; Kuhn et al. 2020; Version 1.4.1717). The yawn morphology (Y_CT_ and Y_UCT_) was the response variable (binomial error distribution, N_observations_ = 319). The fixed factors were the CONTEXT (resting-sleeping/tension), the SEX of the yawner (male/female), the ADI values (dominance rank), and the DAYTIME (8:00–10:00 am; 10:00–12:00 am; 12:00 am–2:00 pm; 2:00–4:00 pm; 4:00–6:00 pm). No collinearity was found between the fixed factors (range VIF_min_ = 1.00; VIF_max_ = 1.10). The yawner identity (ID yawner) was included as a random factor.

For both models (MORPHOLOGY and DURATION), by using a likelihood-ratio test (LRT, Anova with argument test “Chisq”; Dobson 2002), we tested the significance of the full model (Forstmeier and Schielzeth 2011), by comparing it to a control model comprising the random factor (ID yawner) and the fixed factor DAYTIME. Then, the* p* values for the individual predictors were calculated based on likelihood-ratio tests between the full and the null model by using the R-function *Anova* in the R-package *car* 3.0–10 (Fox and Weisberg 2019).

#### The presence of a yawning event is predictive of behavioral shifting (MODEL_Shift_*, *Prediction 

To investigate if the presence of a yawn predicts a behavioral shifting, we ran a generalized linear mixed model (GLMM; *glmmTMB* R-package; Berry et al. 2017; R Core Team, 2020; Version 1.4.1717). We included in the model only those spontaneous yawn events (*N* = 288) that were preceded and followed by at least 90 s of videos in which the animal remained completely visible. This procedure allowed us to match C1 and C2 to each Y slot. The presence/absence of BEHAVIORAL SHIFTING was the response variable (binomial error distribution; N_observations_ = 1020). The fixed factors were the CONDITION (C1, Y, C2, BL) (Figure S2), the SEX of the yawner, the ADI, and MORPHOLOGY. No collinearity was found between the fixed factors (range VIF_min_ = 1.00; VIF_max_ = 1.02).

To understand if BEHAVIORAL SHIFTING happened before or after the yawn, we ran a second GLMM. For this model, we focused on the Yawn slot (Y). The BEHAVIORAL SHIFTING was the response variable (binomial error distribution; N_observations_ = 288). The fixed factors were the Y-CONDITION (30 s preceding and 30 s after the yawn event, t_0_), the SEX of the yawner, the ADI, and the MORPHOLOGY (Y_CT_/Y_UCT_). No collinearity was found between the fixed factors (range VIF_min_ = 1.05; VIF_max_ = 1.09).

For both models, we compared the full model against a control model including the random factor (ID) and SEX, ADI, MORPHOLOGY as control factors.

#### Seeing others’ yawns increases the likelihood of yawn response in the observer (MODEL_response_, Prediction 5)

To investigate the presence of yawn response, we ran a generalized linear mixed model. The presence/absence of yawning response was the response variable (binomial error distribution, N_observations_ = 121). The fixed factors were the SEX COMBINATION between the trigger and the receiver (male–female, female-male, male-male, female-female), the absolute values of ΔADI (|ADI_trigger_—ADI_receiver_|), the MORPHOLOGY (Y_CT_/Y_UCT_), the CONTEXT and the SEEN (yes/no). The interaction between the identity of the trigger and receiver was inserted as a random factor (ID_trigger_*ID_receiver_). No collinearity was found between the fixed factors (range VIF_min_ = 1.17; VIF_max_ = 2.38). By using a likelihood-ratio test (Anova with argument test “Chisq”; Dobson 2002), to test the significance of the full model (Forstmeier and Schielzeth 2011), we compared the full model against a control model comprising all the factors except for the variable SEEN.

## Results

### Prediction 1: Different yawn variants are present in drills

**Fig. 1:**
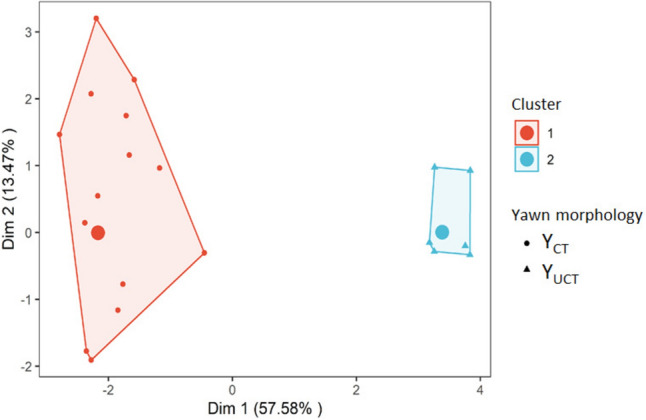
Bi-dimensional representation of the whole sample of yawns obtained by initializing the principal component analysis of mixed data (PCAmix) combined with the k-means clustering results. The* differently shaped points* on the map (*circles* and* triangles*) reflect the a priori classification of Y_CT_ (N = 25) and Y_UCT_ (*N* = 39); in the clouds identified by the k-means clustering,* red* and* blue clusters* represent the distribution of Y_CT_ and Y_UCT_, respectively. Dimension 1 (Dim 1: 57.58%) and Dimension 2 (Dim 2: 13.47%) represent the original variables, a projection or "shadow" of the original data set. Each dimension represents a certain amount of the variation (i.e., information) contained in the original data set

We collected a total of 319 spontaneous yawns (Y_CT_ = 104; Y_UCT_ = 215) from 11 individuals. From this sample, we extracted and codified via FACS both Y_CT_ (*N* = 25, from eight individuals) and Y_UCT_ (*N* = 39, from eight individuals). The a priori identification of Y_CT_ and Y_UCT_ was confirmed by the k-means unsupervised clustering analysis. The data for both Y_CT_ and Y_UCT_ clustered with the percentages of 100% (Fig. 1). The different AUs recorded during the peak of Y_CT_ and Y_UCT_ and the results of the Fisher’s exact test are reported in Table 3.

**Table 3:** Comparison of the Action Units (AU) observed in Y_CT_ (yawn with covered teeth) and Y_UCT_ (yawn with uncovered teeth), the relative *p* values obtained by Fisher’s exact test (significant *p* values in bold)

AU code	AU description	Y_CT_ (%)	Y_UCT_ (%)	*p* values
AU1/2	Brow raiser	2 (8.00)	0 (0.00)	0.149
AU6	Check raiser	2 (8.00)	35 (89.74)	** < 0.001**
AU8	Lips towards each other	7 (28.00)	0 (0.00)	** < 0.001**
AU9 + 10	Nose wrinkle and upper lip raiser	0 (0.00)	39 (100.00)	** < 0.001**
AU12	Lip corner puller	0 (0.00)	39 (100.00)	** < 0.001**
AU16	Lower lip depressor	0 (0.00)	39 (100.00)	** < 0.001**
AU25 + 26	Lips parted and jaw drop	25 (100.00)	0 (0.00)	** < 0.001**
AU25 + 27	Lips parted and mouth stretch	0 (0.00)	39 (100.00)	** < 0.001**
AU43	Closure of the eyes	4 (16.00)	5 (12.82)	0.728
AU45	Blink of the eyes	0 (0.00)	24 (61.54)	** < 0.001**
AD101	Scalp retraction	0 (0.00)	31 (79.49)	** < 0.001**
EAD3	Ears flattener	0 (0.00)	39 (100.00)	** < 0.001**

Y_CT_ (%) = percentage out of a total of 25 Y_CT;_ Y_UCT_ (%) = percentage out of a total of 39 Y_UCT_

### Prediction 2 and 3: Males display shorter yawns (2a) and more Y_UCT_ than females under situations of social tension (2b) OR males display longer yawns (3a) and more Y_UCT_ than females independently from social tension situations (2b)

The full model built to investigate which factors influenced the duration of the yawning event significantly differed from the null model including only the random factor (likelihood-ratio test: χ^2^ = 24.939,* df* = 4, *P* < 0.001; Table 4). We found that male yawns lasted longer than those of females and the Y_UCT_ (mean 3.17 s ± 0.10 SE) lasted longer compared to Y_CT_ (mean 2.56 s ± 0.13 SE) (Table 4, Prediction 2a not supported). No significant effect was found for the dominance rank.

**Table 4:** Estimated parameters (Coeff), standard error (SE), and results of the likelihood-ratio tests (χ^2^) for the spontaneous yawning (*Model*_*duration*_*, Model*_*morphology*_*, Model*_*shift*_)

Prediction 2a OR 3a (LMM)—Males display shorter yawns than females OR longer yawns than females					
Fixed effects	Coeff	SE	χ^2^	*df*	*P*
Tested variables					
Intercept	0.515	0.174	n/a	n/a	n/a
Context	0.075	0.087	0.745	1	0.388
Sex	− 0.319	0.111	8.256	1	**0.004**
ADI	0.229	0.198	1.331	1	0.249
Morphology	0.242	0.063	14.821	1	** < 0.001**
Control variables					
Daytime			6.870	4	0.143
Daytime (10:00–12:00 pm)	0.122	0.071			
Daytime (12:00 am–2:00 pm)	0.069	0.302			
Daytime (2:00–4:00 pm)	0.497	0.238			
Daytime (4:00–6:00 pm)	0.144	0.080			

Prediction 2b OR 3b (GLMM)—Males display more Y_UCT_ than females under situations of social tension OR independently from situations of social tension						
Fixed effects	Coeff		SE	χ^2^	*df*	*P*
Tested variables						
Intercept	1.021		0.572	n/a	n/a	n/a
Context	0.696		0.422	2.727	1	0.099
Sex	− 1.224		0.430	8.099	1	**0.004**
ADI	− 0.106		0.585	0.033	1	0.856
Control variables						
Daytime				1.773	4	0.778
Daytime (10:00–12:00 pm)	− 0.308		0.298			
Daytime (12:00 am-2:00 pm)	− 0.235		1.249			
Daytime (2:00–4:00 pm)	− 0.638		0.955			
Daytime (4:00–6:00 pm)	− 0.006	0.350				

Prediction 4 (GLMM)—The presence of a yawning event predicts a shift					
Fixed effects	Coeff	SE	χ^2^	*df*	*P*
Tested variable					
Intercept	− 0.631	0.507	n/a	n/a	n/a
Condition			144.43	3	** < 0.001**
Y	2.219	0.230			
C2	0.784	0.228			
BL	0.089	0.243			
Control variables					
ADI	− 1.078	0.499	4.669	1	0.031
Sex	0.075	0.374	0.040	1	0.841
Morphology	0.084	0.176	0.233	1	0.632

Prediction 4 (GLMM)—The presence of shift 30 s before or after the yawning event						
Fixed effects	Coeff	SE	χ^2^	*df*	*P*	
Tested variable						
Intercept	− 0.912	0.857	n/a	n/a		n/a
Y- Condition	2.021	0.519	15.148	1		** < 0.001**
Control variables						
ADI	0.346	0.867	0.159	1		0.690
Sex	1.088	0.599	3.300	1		0.069

Significant *P *values are shown in bold

Variance for the random factors: ID_yawner_ = 0.0008 ± 0.028SD. Marginal R^2^ = 0.129; Conditional R^2^ = 0.131; N_observation_ = 319; N_yawner_ = 11

Variance for the random factors: ID_yawner_ = 9.92e^−10^ ± 3.15e^−5^SD. Marginal R^2^ = 0.060; Conditional R^2^ = 0.061; N_observation_ = 319; N_yawner_ = 11

Variance for the random factors: ID_yawner_ = 0.0978 ± 0.3127SD. Marginal R^2^ = 0.200; conditional R^2^ = 0.223; N_observation_ = 288; N_individuals_ = 9

Variance for the random factors: ID_yawner_ = 0.01639 ± 0.128SD. Marginal R^2^ = 0.193; Conditional R^2^ = 0.197; N_observation_ = 288; N_individuals_ = 9

*Df* degree of freedom, *n/a* not applicable

**Fig. 2:**
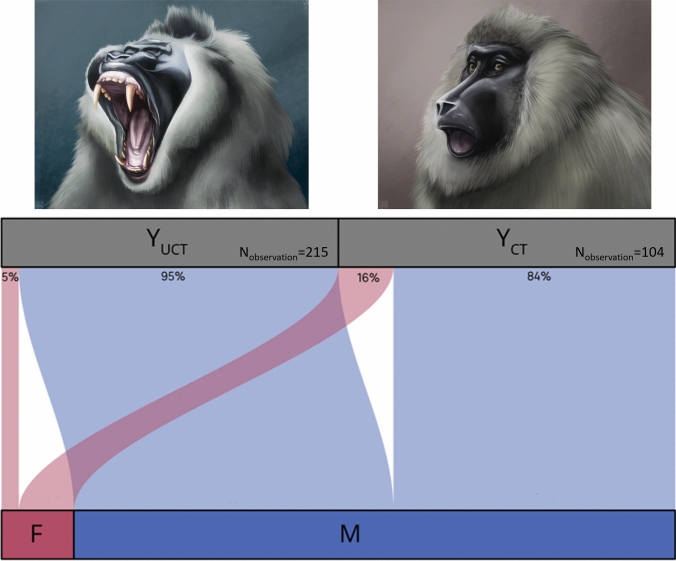
The alluvial plot showing the probability for males and females to perform Y_CT_ (males = 84%, females = 16%) or Y_UCT_ (males = 95%, females = 5%). (R package ‘ggalluvial’; Brunson and Read 2023). N_observations_ = 319; 104 Y_CT_ and 215 Y_UCT_

The full model built to investigate which factors influenced the morphology of the yawning significantly differed from the null model (likelihood-ratio test: χ^2^ = 11.271, df = 3, *P* = 0.010; Table 4). In particular, we found that the SEX of the yawner significantly affected the morphology with males performing more Y_UCT_ than females (Fig. 2, Table 4, Prediction 2b partially supported). The context and the dominance rank had no significant effect on the response variable.

### Prediction 4: The presence of a yawning event is predictive of behavioral shifting

**Fig. 3:**
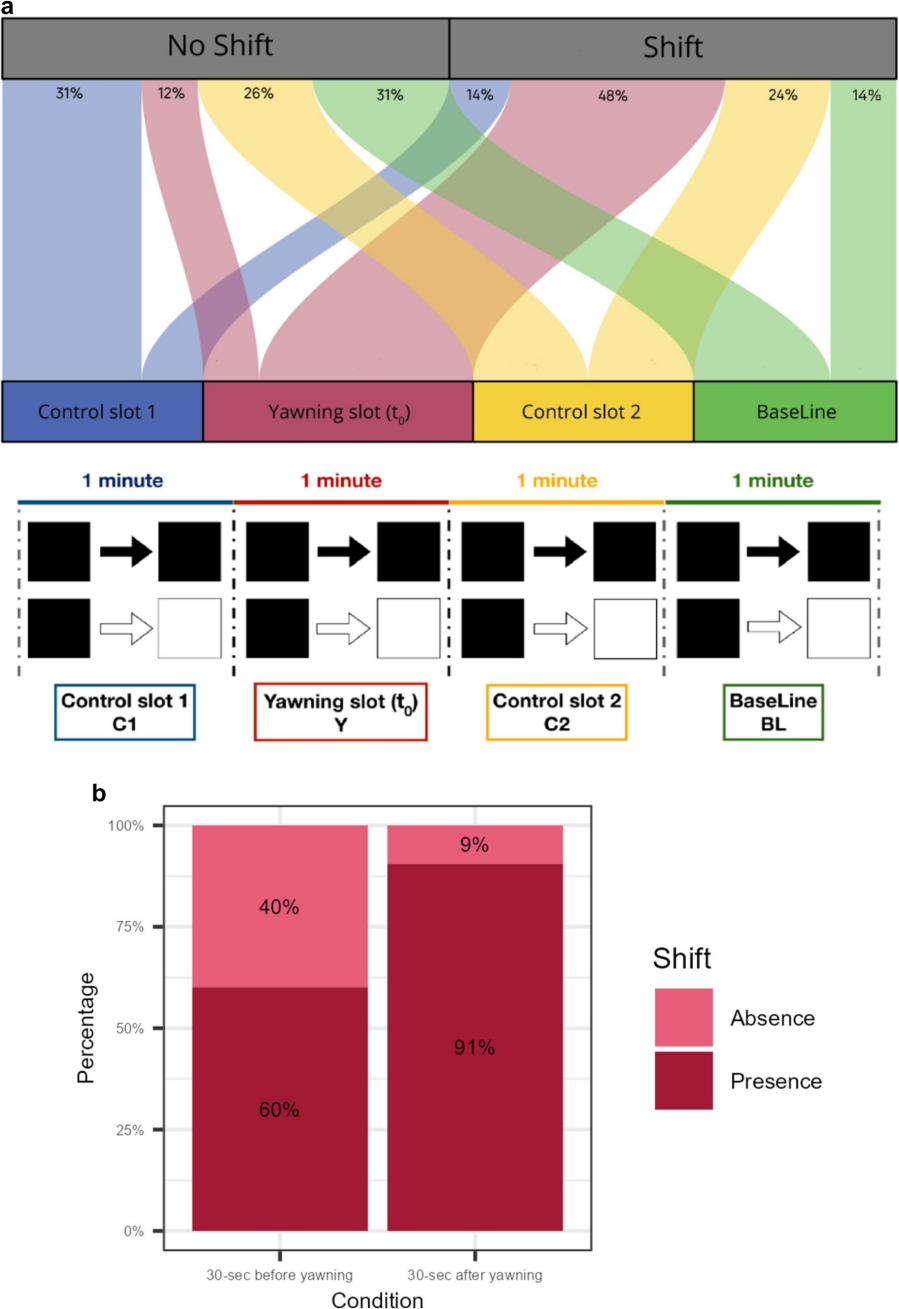
**a** Scheme showing the four different time slots considered in the analysis of the behavioral shifting: 1-min Yawning slot (Y) including the yawning event occurring at t_0_ (*red*); 1-min Control slot (C1) preceding Y (*blue*); 1-min Control slot (C2) following Y (*yellow*); 1-min BaseLine slot (BL) (*green*).* Squares* indicate the behaviors (black square/black square = no shift; black square/white square = yes shift). The upper part of the figure is an Alluvial plot showing the probability of a SHIFTING event in the four conditions (14% in C1,* blue stream*; 48% in SY,* red stream*; 24% in C2,* yellow stream*; 14% in BL,* green stream*). (R package ‘ggalluvial’; Brunson and Read 2020). N_observations_ = 288, N_shift-C1_ = 42; N_shift-Y_ = 146; N_shift-C2_ = 72; N_shift-BL_ = 45.**b** Percentages of presence (*dark red*) and absence (*light red*) of behavioral shifting in the 30 s before the yawning event and in the 30 s after the yawning event. The graph compares the two halves of the Y condition described in caption of **a** N_observations_ = 288

We found a significant difference between the full and the control model (*χ*2 = 144.43, *df* = 3, *P* < 0.001). The variable CONDITION significantly affected the BEHAVIORAL SHIFTING response variable, with the Tukey post hoc test showing that the highest probability for a behavioral shift to occur was during the Y period than during C1, C2, and BL) (t-ratio_C1 vs. Y_ = − 9.653,* df* = inf, *P* < 0.001; t-ratio_C1 vs. C2_ = − 3.287,* df* = inf, *P* = 0.0056; t-ratio_C1 vs. BL_ = − 0.364,* df* = inf, *P* = 0.9835; t-ratio_Y vs. C2_ = 7.049,* df* = inf, *P* < 0.001; t-ratio_SY vs. BL_ = 9.408,* df* = inf, *P* < 0.001; t-ratio_C2 vs. BL_ = 2.942,* df* = inf, *P* = 0.0172). (Fig. 3a, Table 4).

As a second step, we found a significant difference between the full and the control model (*χ*2 = 21.849, *df* = 1, *P* < 0.001). The probability to have a behavioral shifting was higher in the 30 s after the yawn event (Fig. 3b, Table 4).

### Prediction 5: Seeing others’ yawns increases the likelihood of yawn response in the observer

We included in the model only those cases (*N* = 121) for which we could follow the animals, considering both instances where the receiver could see the yawning stimulus and where they could not, for at least 3 min after the occurrence of the previous yawn.

**Fig. 4:**
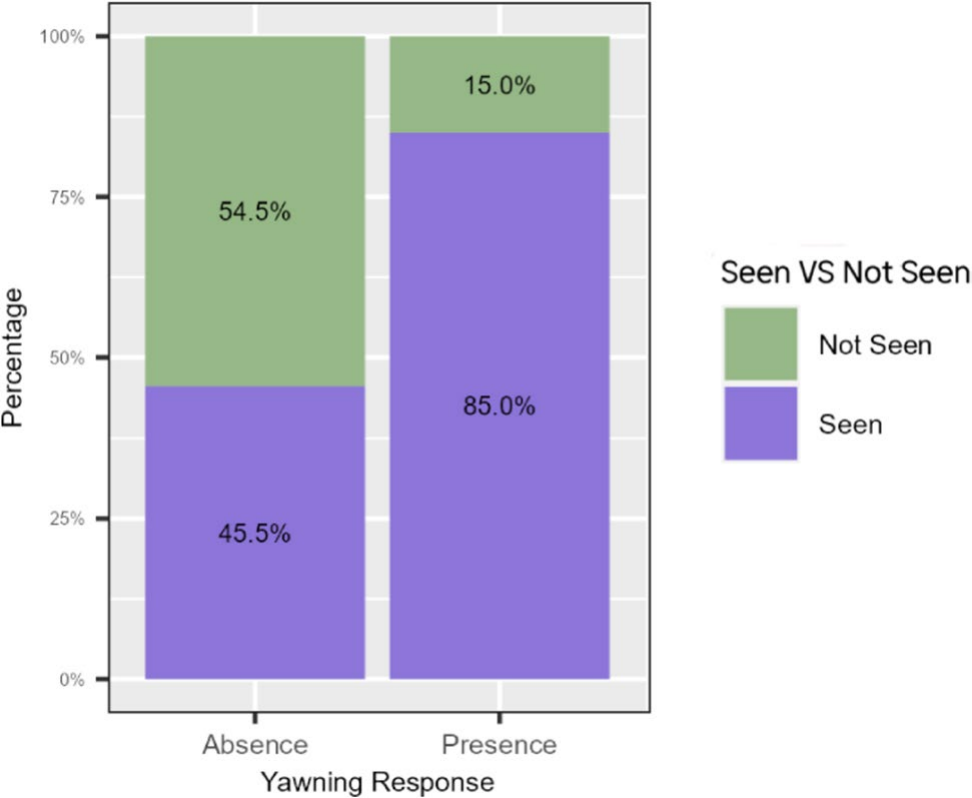
Percentages of absence and presence of yawning response in the conditions SEEN (purple, 63 cases) and NOT SEEN (green, 58 cases). N_observations_ = 121; presence_yawn_ = 20; absence_yawn_ = 101

The full model significantly differed from the control model (likelihood-ratio test: χ^2^ = 10.564,* df* = 2, *P* = 0.005; Table 5). The results show that seeing others’ yawns significantly increased the probability of a yawn response in the receiver within 3 min (Fig. 4). In particular, we obtained that of 63 events in which recipients saw the trigger yawn, recipients responded in 16 cases thus in 25.81% of cases. Of 58 events in which recipients did not seen the yawn only in three cases did they respond by yawning (5.17% of cases). The time latency of the yawning response was 17.13 s ± 7.78 SE.

**Table 5:** Estimated parameters (Coeff), standard error (SE), and results of the likelihood-ratio tests (**χ**^**2**^) of the *MODEL*_*response*_

Prediction 4 (GLMM)—Seeing others’ yawns increases the likelihood of yawn response in the observer							
Fixed effects	Coeff	SE	2.5% CI	97.5% CI	χ^2^	*df*	*P*
Intercept	− 1.440	0.836	− 3.079	0.199	n/a	n/a	n/a
Tested variable							
Seen/Not seen	2.210	0.780	0.680	3.739	8.017	1	**0.005**
Control variables							
Context	− 1.571	1.349	− 4.216	1.074	1.355	1	0.244
Morphology	− 0.635	0.964	− 2.525	1.255	0.433	1	0.510
ADI	0.845	1.364	− 1.829	3.519	0.383	1	0.536
Sexcomb					3.898	2	0.142
Sexcomb1	− 0.706	1.241	− 3.139	− 3.139			
Sexcomb10	− 2.705	1.391	− 1.725	0.021			

Variance for the random factors: ID_receiver_ = 3.355*e-01 ± 5.792*e-01SD; random ID_trigger_ = 1.081*e-09 ± 3.287*e-05SD. Marginal* R*^2^ = 0.053; Conditional* R*^2^ = 0.053; N_observation_ = 121; N_receiver_ = 9; N_trigger_ = 8

Significant *P* values are shown in bold

Df degree of freedom, n/a not applicable

## Discussion

Drills show a certain degree of yawning variability, which is linked to the exposure of teeth (covered, Y_CT_ or uncovered, Y_UCT_) that does not seem to be affected by contextual social factors. We were able to conduct a FACS analysis on a subset of available yawns (25 Y_CT_ and 39 Y_UCT_) and the clusters obtained were totally consistent with the a priori classification (Fig. 1). The morphological difference between Y_CT_ and Y_UCT_ was based on ten out of 12 AUs (Table 3) thus underlining that a number of different AUs concur in differentiating the two morphs of yawns (Prediction 1 supported). Compared to Y_CT_, Y_UCT_ was characterized by a higher number of AUs recruited. Given the AU26 and AU27 are necessarily expressed in association with AU25, but all the other AUs independent from each other, we can reasonably state that the observed differences between Y_CT_ and Y_UCT_ are not a byproduct of the simple larger mouth dropping. Although such an approach has never been applied before to describe the different yawning configurations, our results are in line with the macroscopic descriptions of yawning in other primate species (*Theropithecus gelada*, Leone et al. 2014; *Pan troglodytes*, Vick and Paukner 2010; *Macaca tonkeana* and *M. fuscata*, Zannella et al. 2017, 2021).

After defining the two different yawn morphologies in drills, we tested if Y_CT_ and Y_UCT_ are affected by the sex of the yawner and the context of the performance (relaxed vs. social tension condition). Our findings show that males performed longer yawns (Prediction 2a not supported, Prediction 3a supported) and more Y_UCT_ than females (Fig. 2) independently from the social context (Prediction 2b not supported, Prediction 3b supported). Although our data need to be interpreted with caution due to the small sample size and the disproportionate number of yawns (80%) performed by the two fully grown males, they seem to support the *Brain Cooling Hypothesis* (Gallup et al. 2016; Massen et al. 2021). Due to the high degree of sexual dimorphism in cranial size in drills (Osman-Hill 1970, 1974), the long-lasting and large yawns recorded in males independently from the social context make the *Brain Cooling Hypothesis* the most plausible to explain our results.

We found no effect of social context on either duration or types of yawns (Y_CT_/Y_UCT_) thus leading to reject the *Social Distress Hypothesis*. Specifically, we found that yawn morphology and duration were similar in the relaxed and social tension situations. Moreover, contrary to other monkey species in which the largest yawns were also the shortest ones under tense conditions (Deputte 1994; Zannella et al. 2021), in drills Y_UCT_ lasted longer than Y_CT_ suggesting that the longer duration of this kind of yawns can be a byproduct of the higher number of AUs recruited. The fact that more AUs are recruited in the Y_UCT_, which are more common among the fully grown males, is consistent with the *Brain Cooling Hypothesis*. Subjects with larger brains would require longer and more robust yawns to achieve the same cooling effects (Gallup et al. 2016; Massen et al. 2021).

To explain the absence of any effect of the social context on yawning emission, two interpretations are possible, at the same time, not mutually exclusive. Y_UCT_ is not always linked to the arousal state of the subject and/or, in some cases, intra-group aggressive events are not sufficient to perturb subjects’ affective homeostasis. Therefore, our data suggest that while both Y_CT_ and Y_UCT_ can be related to the intrinsic factors of the animals (e.g., sex), the two different morphs do not always seem to inform about social contingent factors (e.g., intra-group conflict). Clearly, our data cannot be generalized, because a larger sample size could have provided more solid information especially about the possible role of dominance rank in shaping yawning activity that it is difficult to unveil based on our limited number of males.

Yawning, independently from its morphology, has been found to be associated with a behavioral shift of the subjects (Fig. 3a) that changed their behavioral state immediately after yawning (Fig. 3b) (Prediction 4 supported). This finding is consistent with correlational data already found in other non-human primate species. In chimpanzees, for example, yawning correlates with the change in the general activity levels (Vick and Paukner 2010). Similar findings were achieved by the comparison of two sympatric primate Malagasy species. Ring-tailed lemurs (*Lemur catta*)*,* which is characterized by a more dynamic lifestyle, showed higher level of yawning than sifaka (*P. verreauxi*), a folivorous species, which spends a large amount of time resting thus infrequently engaging in behavioral state changes. This correlational evidence led the authors to suggest that the extent of spontaneous yawning can be related to the level of behavioral dynamicity in each lemur species (Zannella et al. 2015).

Yawning in drills was not only found to be associated to a behavioral state change (Fig. 3a) but it preceded such changing, with the shifting mainly occurring in the 30-s time window after the yawning event (Fig. 3b). This finding is also in agreement with the higher probability of behavioral state changing in C2 compared to C1 probably indicating a carryover effect due to the previous yawn. Such predictability effect of yawning suggests that it could be a reliable indicator of the imminent shifting behavior of the yawner. This finding, together with the tendency of drills to respond to others’ yawn (Fig. 4), indicates that yawning can be a vehicle of synchronization of some activities in primates. Similar results have been also obtained for wild social carnivore species such as spotted hyaenas (*Crocuta crocuta*, Casetta et al. 2022) and African lions (*Panthera leo*, Casetta et al. 2021). In these species, which live in fission fusion societies, the maintenance of subgroup cohesion seems to be particularly important due to the need for these carnivore species to cooperate in offspring care, territorial defense and collective hunting (Vullioud et al. 2019; Duranton and Gaunet 2016). In an interesting experimental study, Gallup and Meyers (2021) explored the social role of yawn in our species. The authors found that after perceiving others’ yawns, human subjects increased their vigilance levels and were able to detect more rapidly a negative stimulus present in the environment. This is the first evidence of changes in cognitive performance induced by the simple observation of others’ yawns. Recently, the study has been replicated basically finding the same results (Gallup and Wozny 2022). Although the constraints on generalization make direct comparisons difficult, our data on the immediate effect of spontaneous yawning on subsequent behaviors and the tendency of the observers to replicate others’ yawns (Prediction 5 supported, Fig. 4) indicate that spontaneous and contagion yawning can have a role in group synchronization also in drills. Unfortunately, we have insufficient data to test the immediate effect of the yawning response on a possible increase of joint action between the first and the second yawner. Obviously, to effectively verify both ultimate functions of spontaneous yawning in drills, long-term data coming from wild populations are needed (see Palagi and Bergman 2021 for an extensive review). Ours is only the first attempt to understand which factors are at the basis of the yawning phenomenon and which effects it can produce at group level. However, given our strict protocol, we believe that the study can be replicated not only in other captive and wild groups of drills but also in other non-primate and primate species. This will help understand whether our results strictly depend on the characteristics of the study group or they can be generalized.

### Supplementary Information

Below is the link to the electronic supplementary material.

Supplementary file1 Fig. S1 Screenshots showing the two yawn variants in juveniles, females and males: (A1) juvenile YCT and (A2) juvenile YUCT; (B1) female YCT and (B2) female YUCT; (C1) male YCT and (C2) male YUCT (TIFF 2304 KB)

Supplementary file2 The scheme illustrates the four different time slots considered in the analysis of the behavioral shifting: 1-min Yawning slot (Y) including the yawning event occurring at t0 (red); 1-min Control slot (C1) preceding Y (blue); 1-min Control slot (C2) following Y (yellow); 1-min BaseLine slot (BL) (green). Squares indicate the behaviors (black square/black square=no shift; black square/white square=yes shift) (TIF 243 KB)

Supplementary file3 (CSV 2 KB)

Supplementary file4 (CSV 20 KB)

Supplementary file5 (CSV 7 KB)

Supplementary file6 (CSV 5 KB)

## Data Availability

The data that support the findings of this study are available as Supplementary Material.
