# Supplementary figures and images for: More than a simple fixed action pattern: Yawning in drills
Source: Primates. 2024 Apr 22;65(4):281–97. doi: 10.1007/s10329-024-01127-7 (PMC11219445; doi:10.1007/s10329-024-01127-7)

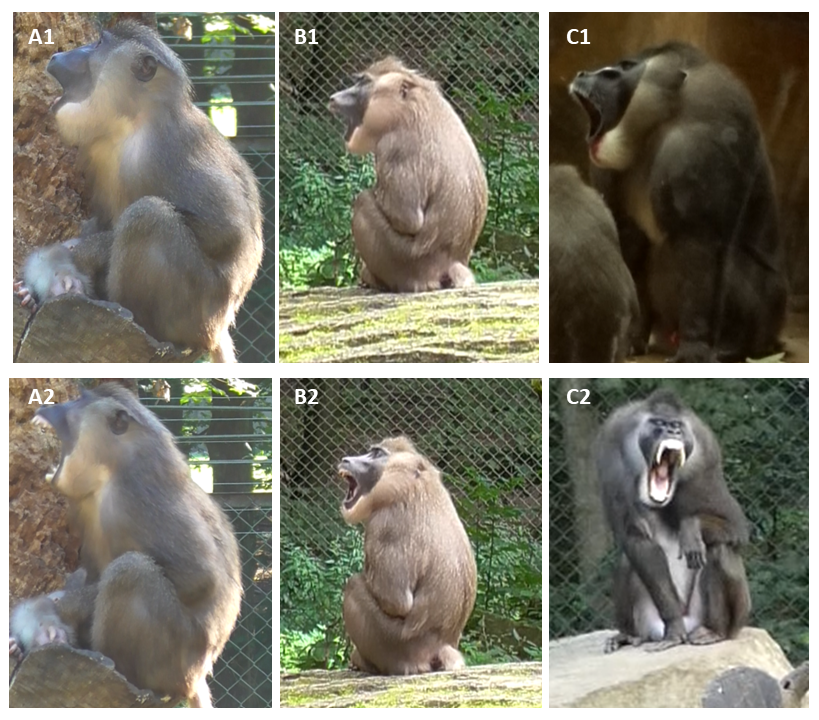

Supplement: Supplementary file 1 — Supplementary file1 Fig. S1 Screenshots showing the two yawn variants in juveniles, females and males: (A1) juvenile YCT and (A2) juvenile YUCT; (B1) female YCT and (B2) female YUCT; (C1) male YCT and (C2) male YUCT (TIFF 2304 KB) [file 10329_2024_1127_MOESM1_ESM.tiff]

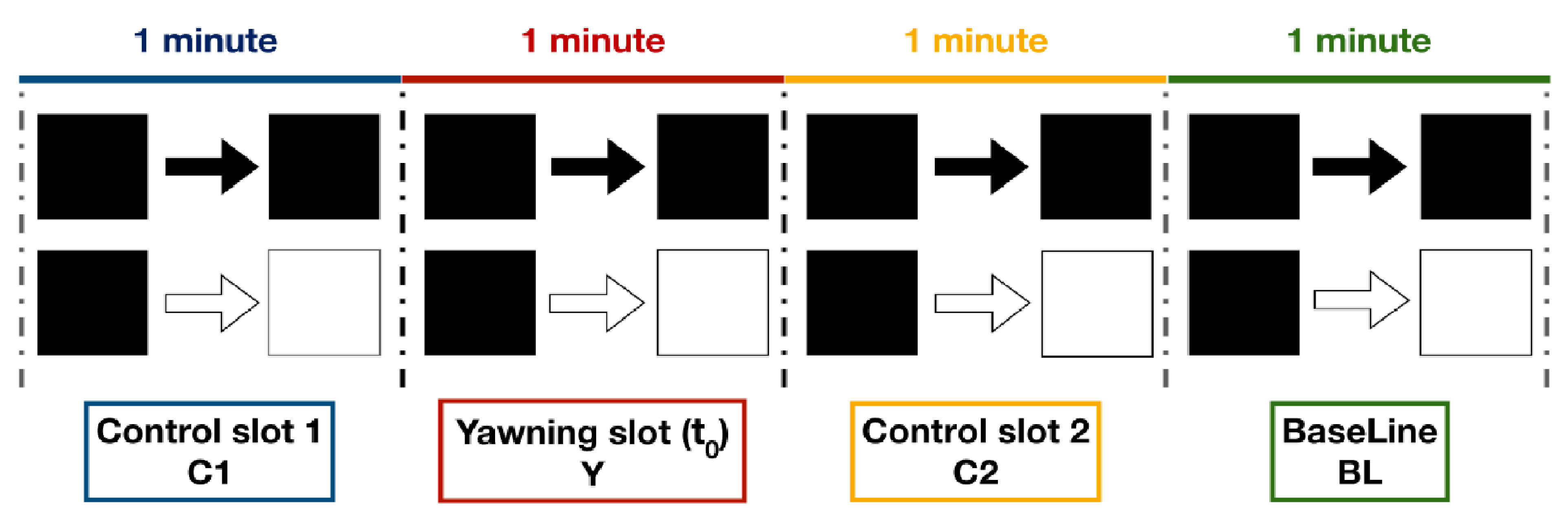

Supplement: Supplementary file 2 — Supplementary file2 The scheme illustrates the four different time slots considered in the analysis of the behavioral shifting: 1-min Yawning slot (Y) including the yawning event occurring at t0 (red); 1-min Control slot (C1) preceding Y (blue); 1-min Control slot (C2) following Y (yellow); 1-min BaseLine slot (BL) (green). Squares indicate the behaviors (black square/black square=no shift; black square/white square=yes shift) (TIF 243 KB) [file 10329_2024_1127_MOESM2_ESM.tif]
